# Oncogenic signaling by Kit tyrosine kinase occurs selectively on the Golgi apparatus in gastrointestinal stromal tumors

**DOI:** 10.1038/onc.2016.519

**Published:** 2017-02-13

**Authors:** Y Obata, K Horikawa, T Takahashi, Y Akieda, M Tsujimoto, J A Fletcher, H Esumi, T Nishida, R Abe

**Affiliations:** 1 Division of Immunobiology, Research Institute for Biomedical Sciences, Tokyo University of Science, Noda, Chiba, Japan; 2 Department of Surgery, Osaka University, Graduate School of Medicine, Suita, Osaka, Japan; 3 Department of Diagnostic Pathology, Osaka Police Hospital, Osaka, Osaka, Japan; 4 Department of Pathology, Brigham and Women's Hospital and Harvard Medical School, Boston, MA, USA; 5 Division of Clinical Research, Research Institute for Biomedical Sciences, Tokyo University of Science, Noda, Chiba, Japan; 6 National Cancer Center Hospital, Chuo-ku, Tokyo, Japan

## Abstract

Gastrointestinal stromal tumors (GISTs) are caused by gain-of-function mutations in the Kit receptor tyrosine kinase. Most primary GIST patients respond to the Kit inhibitor imatinib, but this drug often becomes ineffective because of secondary mutations in the Kit kinase domain. The characteristic intracellular accumulation of imatinib-sensitive and -resistant Kit protein is well documented, but its relationship to oncogenic signaling remains unknown. Here, we show that in cancer tissue from primary GIST patients as well as in cell lines, mutant Kit accumulates on the Golgi apparatus, whereas normal Kit localizes to the plasma membrane (PM). In imatinib-resistant GIST with a secondary Kit mutation, Kit localizes predominantly on the Golgi apparatus. Both imatinib-sensitive and imatinib-resistant Kit (Kit(mut)) become fully auto-phosphorylated only on the Golgi and only if in a complex-glycosylated form. Kit(mut) accumulates on the Golgi during the early secretory pathway, but not after endocytosis. The aberrant kinase activity of Kit(mut) prevents its export from the Golgi to the PM. Furthermore, Kit(mut) on the Golgi signals and activates the phosphatidylinositol 3-kinase–Akt (PI3K–Akt) pathway, signal transducer and activator of transcription 5 (STAT5), and the Mek–Erk pathway. Blocking the biosynthetic transport of Kit(mut) to the Golgi from the endoplasmic reticulum inhibits oncogenic signaling. PM localization of Kit(mut) is not required for its signaling. Activation of Src-family tyrosine kinases on the Golgi is essential for oncogenic Kit signaling. These results suggest that the Golgi apparatus serves as a platform for oncogenic Kit signaling. Our study demonstrates that Kit(mut)’s pathogenicity is related to its mis-localization, and may offer a new strategy for treating imatinib-resistant GISTs.

## Introduction

Kit, a cell-surface receptor for stem cell factor, belongs to the type III receptor tyrosine kinase (RTK) family that includes platelet-derived growth factor receptor α/β (PDGFRα/β), Flt3 and Fms.^1, 2, 3^ Kit is expressed on interstitial cells of Cajal (ICC), mast cells, hematopoietic cells, germ cells and melanocytes.^4, 5, 6^

Kit is composed of the amino-terminal extracellular portion that binds stem cell factor, a transmembrane domain, and the carboxy-terminal intracellular tyrosine kinase domain.^4, 7^ The binding of stem cell factor autophosphorylates Kit on specific tyrosine residues, for example, Tyr568, Tyr570, Tyr703 and Tyr721.^4, 8, 9^ Kit then binds to other cytoplasmic proteins, and this complex phosphorylates other proteins.^3, 4, 8^ This activates the PI3K–Akt pathway, the Ras–Mek–Erk cascade and Src kinases, which regulate gene expression and cytoskeletal structures, resulting in cell proliferation and survival.^7, 8, 9, 10^

In many gastrointestinal stromal tumors (GISTs) (~85%) and mastocytomas, *Kit* has gain-of-function mutations, causing ligand-independent auto-activation of the receptor^11, 12, 13, 14^ (Supplementary Figure S1a). Mutant Kit (Kit(mut)) transforms a precursor of ICC through permanent activation of the PI3K–Akt pathway, STATs and Erk resulting in development of GIST.^15, 16, 17, 18, 19, 20, 21^ GIST cells can then proliferate autonomously due to the anti-apoptotic effect and cell cycle progression by Kit signals.^17, 18, 19, 20, 21^ PDGFRα(mut) also causes GIST (~5%) in this way.^22, 23^ Ten percent of GISTs have no mutation either in *Kit* or *PDGFRα*.^23, 24^

The drug imatinib, a selective inhibitor of Kit, improved the prognosis of GIST patients and the median overall survival is now estimated more than 5 years.^23, 24, 25^ However, resistance to the drug appears with prolonged use and has become a serious problem.^23, 24^ Most imatinib-resistant cases have a secondary Kit mutation in the kinase domain, and then lose sensitivity to the drug.^24, 26, 27^ Further understanding of oncogenic signals is required for establishment of effective targeting of therapy.

The characteristic intracellular accumulation of Kit(mut) and PDGFRα(mut) in GIST patients is well documented,^28, 29, 30, 31, 32, 33^ but its relationship to oncogenic signaling remains unknown. We recently reported that in mastocytomas, Kit(mut) causes oncogenic signaling on intracellular compartments, such as endo/lysosomes.^34^ We then explored how and where oncogenic Kit signals occur in GISTs. Here we show that in primary GIST patients and cell lines, mutant Kit accumulates on the Golgi apparatus in a manner that depends on its kinase activity. In imatinib-resistant GIST with a secondary Kit mutation, Kit localizes predominantly on the Golgi. Both imatinib-sensitive and imatinib-resistant Kit (Kit(mut)) become fully auto-phosphorylated only on the Golgi and only if in a complex-glycosylated form. Furthermore, Kit(mut) on the Golgi signals and activates the PI3K–Akt pathway, STAT5, and the Mek–Erk pathway. Blocking Kit’s localization to the Golgi from the endoplasmic reticulum (ER) inhibits oncogenic signaling. Plasma membrane (PM) localization of Kit(mut) is not required for its signaling. Src-family tyrosine kinases (SFKs) on the Golgi are essential for oncogenic Kit signaling. Our study demonstrates that Kit(mut)’s pathogenicity is related to its mis-localization, and may offer a new strategy for treating imatinib-resistant GISTs.

## Results

### In cancer tissue from GIST patients, Kit(mut) accumulates on the Golgi apparatus

**Figure 1:**
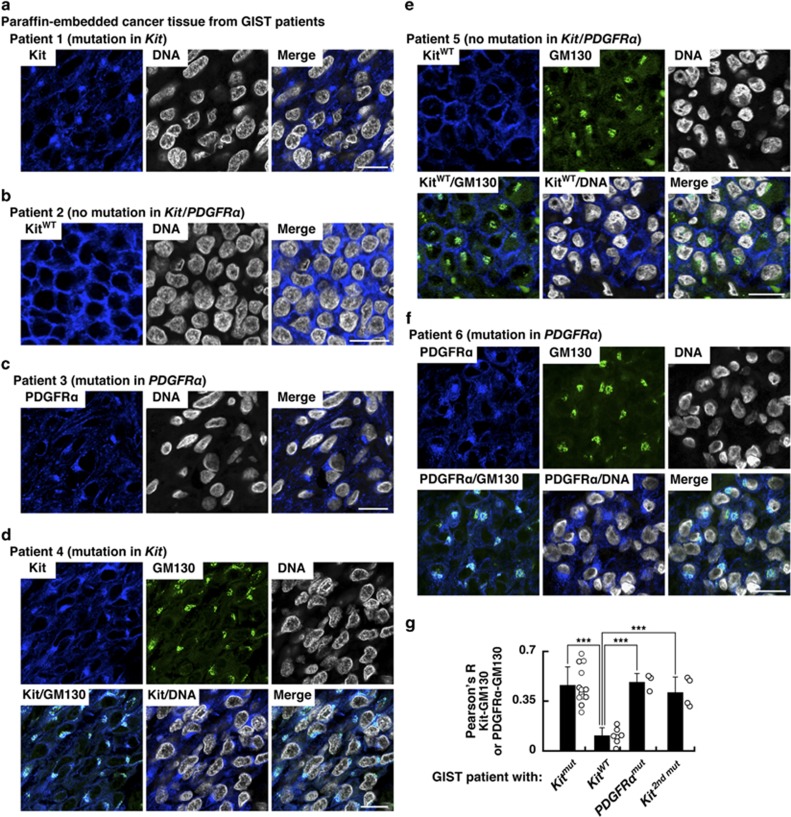
In cancer tissue from GIST patients, Kit(mut) accumulates on the Golgi apparatus. (**a**–**f**) Paraffin-embedded tumor tissue specimens were immuno-stained with anti-Kit (**a**, **b**, **d** and **e**; blue), anti-PDGFRα (**c** and **f**; blue), anti-GM130 (Golgi matrix protein 130 kDa; green), and Höechst33342 (DNA; white). Bars, 20 μm. (**g**) The graph shows Pearson’s *R* correlation coefficients (Pearson’s *R*) between GM130 vs Kit or PDGFRα. Results are means±s.d. (*n*=3–13). ***P<0.001.

To investigate the sub-cellular localization of Kit, we performed confocal immunofluorescence microscopic analyses of cancer tissue from GIST patients. In GIST expressing Kit(mut), Kit accumulated at the perinuclear region (Figure 1a). In contrast, in GIST with no *Kit* mutations or *PDGFRα* mutations, wild-type (wt) Kit localized preferentially at the PM (Figure 1b). Moreover, PDGFRα(mut) localized to the perinuclear region instead of the PM (Figure 1c), indicating that in GIST, type III RTKs with mutations mis-localize. Since the perinuclear region may connect to the Golgi apparatus,^28, 29, 30, 31, 32, 33, 35^ we also stained with a Golgi marker GM130 to see if Kit and PDGFRα were located there. Kit(mut) and PDGFRα(mut), but not Kit(wt), co-localized precisely with GM130 (Figures 1d–f). By calculating Pearson’s *R* correlation coefficients (Pearson’s *R*) between GM130 and the receptors, we found that Kit(mut) and PDGFRα(mut) localized significantly on the Golgi, whereas Kit(wt) did not (Figure 1g and Table 1). Furthermore, in imatinib-resistant GISTs with a secondary Kit mutation, Kit also localized at the Golgi (Figure 1g, Supplementary Figure S1b, and Table 1). Taken together, these results suggest that the oncogenic signaling may occur on the Golgi apparatus.

### In GIST cell lines, Kit(mut) localizes preferentially on the Golgi apparatus

**Figure 2:**
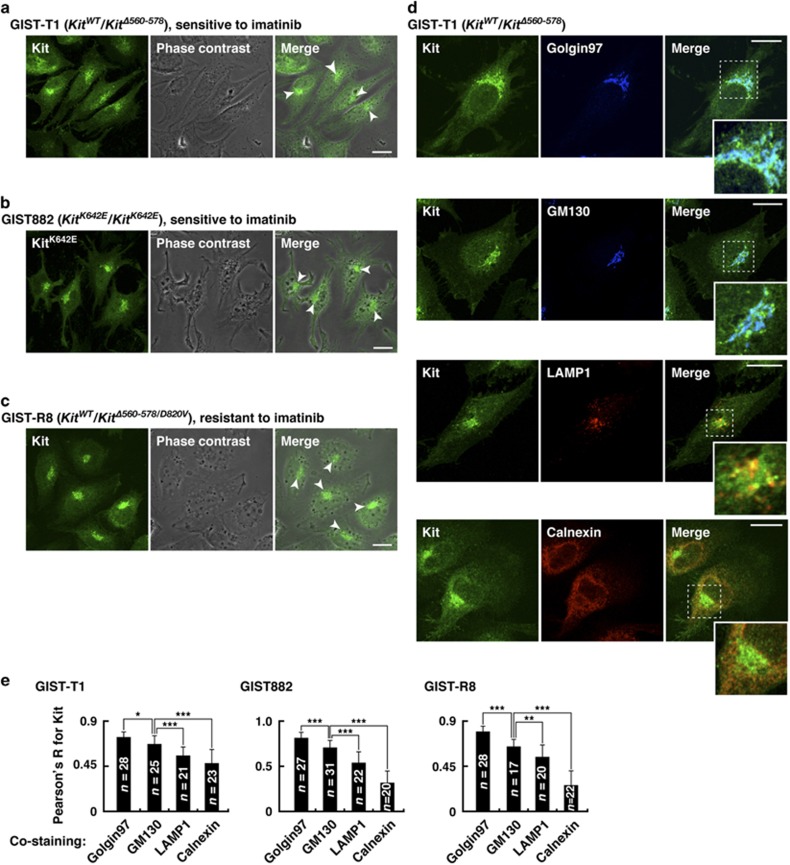
In GIST cell lines, Kit(mut) localizes preferentially on the Golgi apparatus. (**a**–**c**) GIST-T1 (**a**), GIST882 (**b**) and GIST-R8 (**c**) were immuno-stained with anti-Kit. Phase contrast images are shown. Arrowheads indicate the perinuclear region. Bars, 20 μm. (**d**) GIST-T1 cells were double-stained with anti-Kit plus the indicated antibody. Insets show magnified images of the boxed areas. Bars, 20 μm. Golgin97 (*trans*-Golgi marker), blue; GM130, (*cis*-Golgi marker), blue; calnexin (ER marker), red; LAMP1 (lysosome-associated membrane protein 1, endo/lysosome marker), red. (**e**) Pearson’s *R* correlation coefficients were calculated by intensity analysis of Kit vs organelle markers. Results are means±s.d. (*n*=17–31). *P<0.05, **P<0.01, ***P<0.001.

Next, to examine more broadly the sub-cellular localization of Kit, we stained GIST-T1, GIST882 and GIST-R8 cell lines, which endogenously express Kit(mut). GIST-T1 heterozygously expresses a *Kit* exon11 mutant Kit(Δ560–578), whereas GIST882 homozygously expresses Kit(K642E) (Supplementary Figure S2a).^20, 36^ GIST-R8 was established from GIST-T1 by continuous imatinib treatment, and this endogenously expresses Kit(Δ560–578/D820V) that confers imatinib resistance^37^ (Supplementary Figure S2a, bottom). In these cell lines, Kit localized mainly to the perinuclear region but not the PM (Figures 2a–c, arrowheads). A green fluorescent protein-tagged Kit(wt) (Kit(wt)-GFP) was seen predominantly at the PM in HeLa and GIST-T1 (Supplementary Figure S2b), indicating that Kit(mut) distributes specifically to the perinuclear region in GIST cell lines. These results are consistent with those from tumor tissue (Figure 1 and Supplementary Figure S1b).^28, 29, 30^

Next, we stained for Kit in conjunction with golgin97 (*trans*-Golgi marker), GM130 (*cis*-Golgi marker), calnexin (ER marker), LAMP1 (endo/lysosome marker) or LysoTracker (lysosome marker). In GIST cells, Kit co-localized mainly with the Golgi markers (Figure 2d and Supplementary Figures S2c–e). Furthermore, Kit(mut) more precisely localized to the *trans*-Golgi rather than to the *cis*-Golgi (Figure 2e), indicating that in GIST, Kit(mut) accumulates on the *trans*-Golgi cisternae.

### In GIST cells, activation of Kit(mut) occurs mainly on the Golgi apparatus

**Figure 3:**
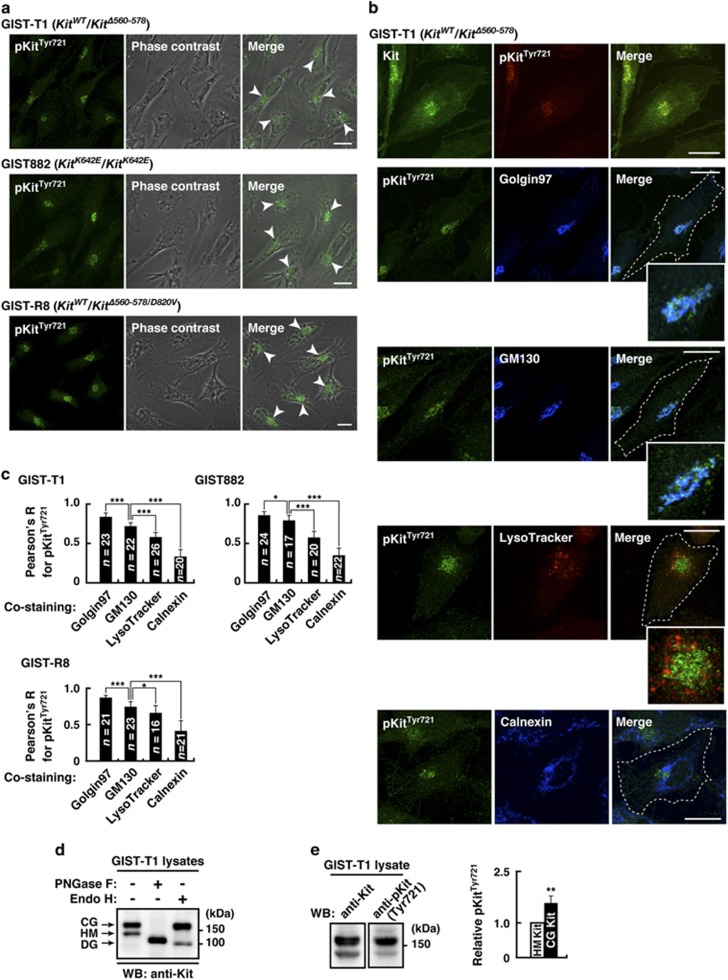
In GIST cells, Kit’s autophosphorylation occurs mainly on the Golgi apparatus. (**a**) GIST-T1, GIST882 and GIST-R8 were immuno-stained with anti-pKit^Tyr721^. Phase contrast images are shown. Arrowheads indicate the perinuclear region. Bars, 20 μm. pKit^Tyr721^, phosphorylation at Tyr721 in Kit. (**b**) GIST-T1 cells were stained with anti-pKit^Tyr721^ in conjunction with the indicated antibody. Golgin97 (*trans*-Golgi marker), blue; GM130, (*cis*-Golgi marker), blue; calnexin (ER marker), blue. Lysosomes were visualized with LysoTracker Red. Insets show magnified images of the perinuclear region. Dashed lines indicate cell borders. Bars, 20 μm. (**c**) Pearson’s *R* correlation coefficients were calculated from intensity analysis of pKit^Tyr721^ vs organelle markers. Results are means±s.d. (*n*=16–26). *P<0.05, ***P<0.001. (**d**) Lysates from GIST-T1 were treated with peptide N-glycosidase F (PNGase F) or endoglycosidase H (endo H) then immunoblotted. CG, complex-glycosylated form; HM, high mannose form; DG, deglycosylated form. (**e**) Levels of phosphorylation of the complex-glycosylated form of Kit are expressed relative to those of the high mannose form of Kit. Results are means±s.d. (*n*=3). **P<0.01.

To examine the relationship between Golgi localization of Kit(mut) and its activity, we immuno-stained for phospho-tyrosine-721 in the kinase domain (pKit[Tyr721]) that indicates Kit’s activation.^7, 8, 9^ Interestingly, pKit[Tyr721] was found only in the perinuclear region in GIST-T1, GIST882 and GIST-R8 (Figure 3a, arrowheads). Furthermore, pKit[Tyr721] distributed significantly to the Golgi rather than other perinuclear compartments, such as lysosomes (Figure 3b and Supplementary Figure S3a). Figure 3c shows that pKit[Tyr721] co-localized with golgin97 (*trans*-Golgi marker) rather than with GM130 (*cis*-Golgi marker), indicating that more activation occurs on *trans-*Golgi than on *cis*-Golgi. Also, tyrosine phosphorylation signals are concentrated in the Golgi (Supplementary Figures S3b and c), indicating that Golgi apparatus serves as a platform for tyrosine phosphorylation signaling in GISTs. In GIST-T1 and HeLa cells transfected with a mastocytoma-type mutant Kit(D814Y),^14, 34, 38^ autophosphorylation of the mutant mainly occurs on the Golgi (Supplementary Figure S3d). This indicates that in these cells, gain-of-function mutations of Kit cause its activation selectively on the Golgi apparatus.

To test Kit’s glycosylation state, we treated Kit from GIST cells with endoglycosidase H, which digests immature high-mannose forms, but not mature complex-glycosylated forms. Most Kit was in a complex-glycosylated form (Figure 3d and Supplementary Figure S3e). Kit shifted to a non-glycosylated form following the complete digestion of N-linked glycans by peptide-N-glycosidase F. Moreover, autophosphorylation of mature Kit was greater than that of immature Kit (Figure 3e and Supplementary Figure S3f). Since mature glycosylated proteins only exist on the *trans*-Golgi, these results suggest that full activation of Kit(mut) occurs on the *trans*-Golgi, and that Kit must be in the complex-glycosylated form to become fully activated.

### Kit(mut) accumulates on the Golgi during the early secretory pathway but not after endocytosis in a manner that depends on its kinase activity

**Figure 4:**
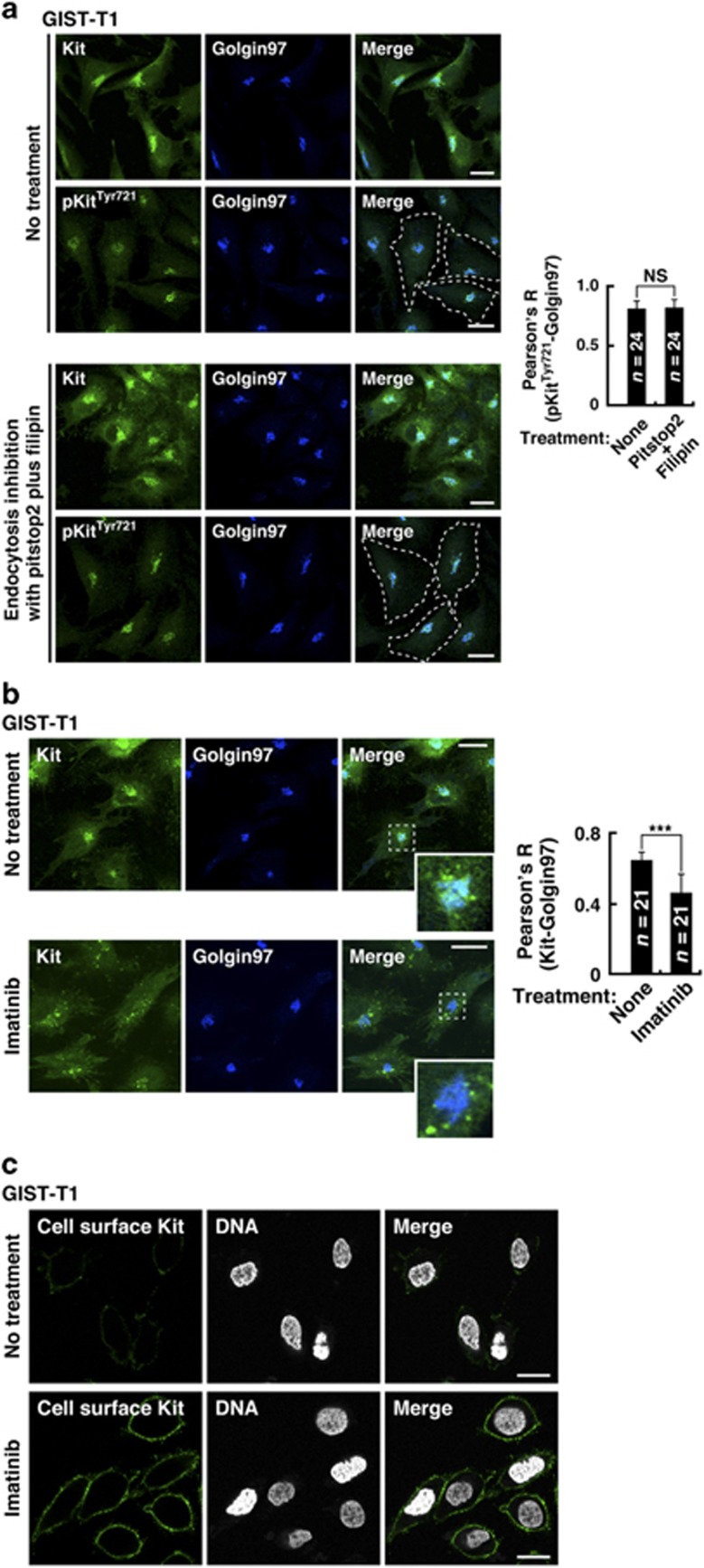
Kit(mut) accumulates on the Golgi during the early secretory pathway but not after endocytosis in a manner that depends on its kinase activity. (**a**) GIST-T1 cells were treated with 50 μM pitstop2 plus 1 μg/ml filipin for 24 h to block endocytosis, then immuno-stained with anti-Kit (green), anti-pKit^Tyr721^ (green) and anti-golgin97 (Golgi marker, green). Dashed lines indicate cell borders. Bars, 20 μm. The graph shows Pearson’s *R* between pKit^Tyr721^ and golgin97. Results are means±s.d. (*n*=24). NS, not significant. *P*=0.68. (**b**, **c**) GIST-T1 cells were treated with 200 nM imatinib (Kit inhibitor) for 4 h. (**b**) Cells were stained for Kit (green) and golgin97 (blue). Insets show magnified images of the boxed areas. Bars, 20 μm. The graph shows Pearson’s *R* between Kit and golgin97. Results are means±s.d. (*n*=21). ***P<0.001. (**c**) Cells stained for the Kit extracellular domain (cell surface Kit, green) and DNA (white) without permeabilization. Bars, 20 μm.

**Figure 5:**
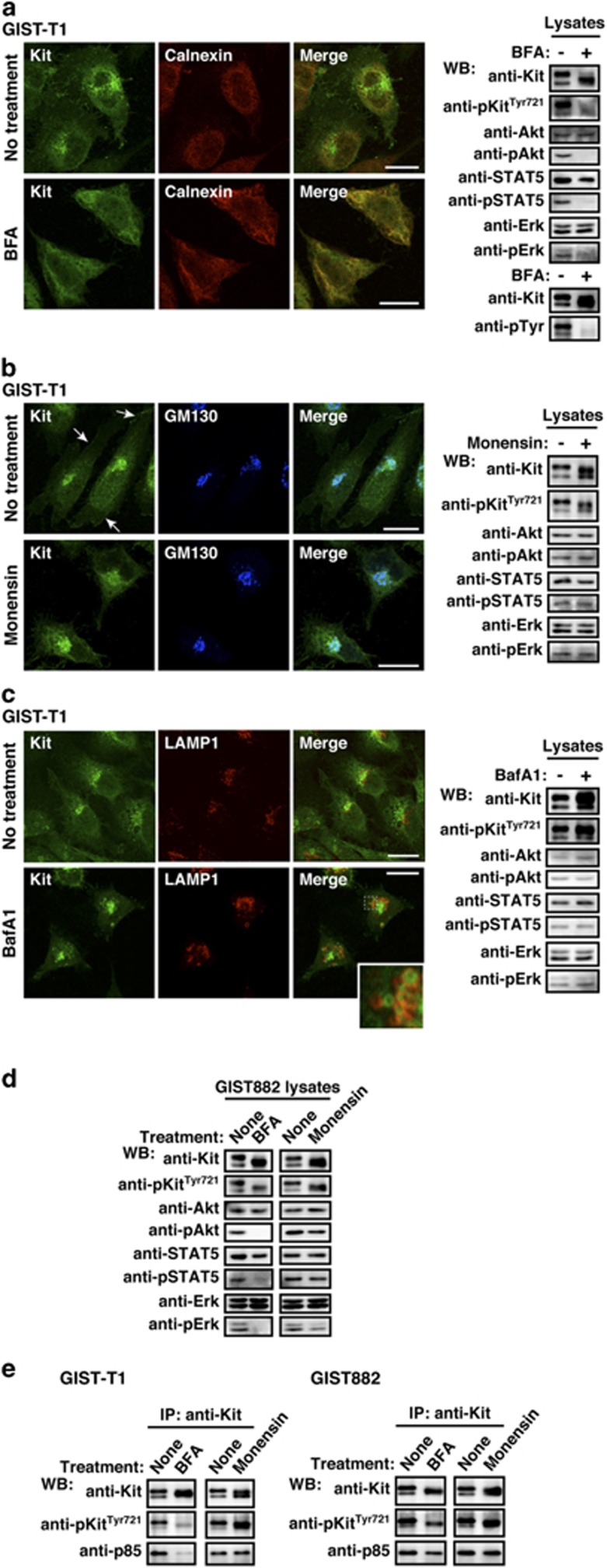
Kit(mut) on the Golgi signals and activates the PI3K-Akt pathway, STAT5, and Erk. (**a**–**c**) GIST-T1 cells were treated with (**a**) 1 μM BFA (brefeldin A; blocks ER export to the Golgi) for 16 h, (**b**) 250 nM monensin (blocks Golgi export) for 24 h, or (**c**) 100 nM bafilomycin A1 (BafA1; blocks endo/lysosomal trafficking) for 24 h. Cells were stained with anti-Kit (green) in conjunction with anti-calnexin (ER marker, red), anti-GM130 (Golgi marker, blue), or anti-LAMP1 (endo/lysosome marker, red). Arrows indicate the PM region. An inset shows a magnified image of the boxed area. Bars, 20 μm. Immunoblots are shown. Phosphorylated proteins are presented as pKit, pAkt, pSTAT5, and pErk. (**d**) GIST882 cells were treated with 1 μM BFA for 16 h or 250 nM monensin for 24 h. Lysates were immunoblotted. (**e**) GIST-T1 and GIST882 were treated with 1 μM BFA for 16 h or 250 nM monensin for 24 h. Anti-Kit immunoprecipitates were immunoblotted.

Next, we examined whether Kit accumulated on the Golgi after the early secretory pathway or after endocytosis from the PM. Recently, we reported that endocytosis of Kit(mut) can be blocked by pitstop2 and filipin, which inhibit clathrin-mediated endocytosis and non-clathrin endocytosis, respectively.^34, 39, 40^ In GIST-T1, endocytosis inhibition for 24 h did not affect either the Golgi localization of Kit or its phosphorylation (Figure 4a), indicating that the major pathway is early secretion. In support of this, Kit’s activation on the Golgi was unaffected by 24-h treatment with monensin, an inhibitor of Golgi export^34, 41^ (Supplementary Figure S4; see also Figure 5b, left panels). These results indicate that Kit(mut) accumulated on the Golgi during the early secretory pathway, but not after endocytosis.

We next examined the role of Kit’s activation in Kit’s localization. To test this, we treated a selective Kit inhibitor imatinib^25, 42^ (Supplementary Figure S5a). In GIST-T1 treated with 200 nM imatinib for 4 h, Kit dispersed from the Golgi region significantly (Figure 4b). Immunostaining of cell surface showed that Kit moved to the PM (Figure 4c). Imatinib, however, did not affect the localization of Kit in GIST-R8, (Supplementary Figures S5b and c). To block the activation of Kit(mut) in GIST-R8, we treated with another Kit inhibitor PKC412^34, 43, 45^ (Supplementary Figure S5b). In GIST-R8, PKC412 significantly decreased the Golgi localization of Kit (Supplementary Figure S5c). Taken together, these results suggest that what prevents Kit’s export from the Golgi is Kit’s kinase activity.

### In GIST, Kit(mut) on the Golgi activates the PI3K–Akt pathway, STAT5 and Erk

Previous reports showed that Kit(mut) activates the PI3K–Akt pathway, STAT5 and the Mek–Erk pathway through phosphorylation^15, 16, 17, 18, 19, 20, 21^ (Supplementary Figures S6a–c), leading to autonomous proliferation of GIST cells. Thus, we examined whether Kit(mut) must localize to the Golgi to activate the PI3K–Akt pathway, STAT5 and Erk. To answer this we inhibited intracellular trafficking. First, we treated GIST-T1 with brefeldin A (BFA), an inhibitor of protein trafficking from the ER to the Golgi.^34, 46^ After 16-h treatment, partially glycosylated Kit co-localized with the ER marker calnexin, a sign of inhibition of trafficking to the Golgi (Figure 5a). Interestingly, pKit[Tyr721] was greatly reduced in BFA-treated GIST-T1, thus Kit could not activate the PI3K–Akt pathway, STAT5, or Erk (Figure 5a, right panels; see also Figure 5e, left panels).

Furthermore, we treated for 24 h with monensin, an inhibitor of protein export from the Golgi apparatus.^34, 41^ Kit(mut) remained phosphorylated and activated downstream molecules, but shifted to a slightly lower molecular weight form (Figure 5b, right; see also Supplementary Figure S4). This form was endoglycosidase H-resistant (Supplementary Figure S6d), indicating that most Kit reached the *trans*-Golgi cisternae. Since monensin inhibits Kit trafficking from the Golgi to the PM (Figure 5b, left images), PM localization of Kit is not required for oncogenic signaling, and Golgi-localization is sufficient.

We previously reported that bafilomycin A1 (BafA1), an inhibitor of endo/lysosomal trafficking, suppresses Akt activation by Kit(mut) in mastocytoma.^34^ Thus, we examined the effect of inhibition of endo/lysosomal trafficking on oncogenic Kit signaling in GISTs. As shown in Figure 5c, BafA1 increased Kit on vesicular structures, suggesting that some Kit traffics along the endo/lysosomal pathway for degradation. Inhibition of endo/lysosomal trafficking, however, does not affect oncogenic Kit signaling (Figure 5c and Supplementary Figure S6e). These results are different from how Kit(mut) in mastocytoma activates Akt and STAT5.

Data from GIST882 and GIST-R8 treated with BFA or monensin strongly supported our idea that in GISTs, oncogenic Kit signals originate from the Golgi (Figure 5d and Supplementary Figure S6f). In GIST882 and GIST-R8, but not GIST-T1, Kit(mut) in the ER became Tyr721 phosphorylated (Figure 5d and Supplementary Figure S6f). Since phosphorylated Kit^Tyr721^ is critical for association with a PI3K p85 subunit,^4, 8, 9^ we tested whether Kit bound to p85 in the ER. Co-immunoprecipitation experiments, following BFA treatment to block ER export, showed that in GIST882 and GIST-R8, Kit still associated with p85, but in GIST-T1 it did not (Figure 5e and Supplementary Figure S6g). In all cases, downstream signaling was abolished. These results indicate that Kit(mut) in the ER is unable to activate Akt, even if Kit(mut) binds to PI3K. In these cell lines, monensin did not affect the Kit–PI3K association (Figure 5e and Supplementary Figure S6g), supporting our finding that Kit(mut) activates Akt signaling through PI3K on the Golgi apparatus.

Supplementary Figure S6h shows that in GIST882’s Golgi, Kit was phosphorylated not only on Tyr721 but also on Tyr568/570 and Tyr703.^4, 8, 47^ In the ER, Kit was dephosphorylated on Tyr568/570 and Tyr703 markedly, although Tyr721 was dephosphorylated partially (Supplementary Figure S6i; compare with Figure 5d). This confirms that full activation of Kit(mut) occurs only after it reaches the Golgi apparatus. In addition, these results suggest that different tyrosines get phosphorylated in different organelles.

Swainsonine inhibits α-mannosidase II at the *medial*-Golgi, altering protein glycosylation.^48^ In swainsonine-treated cells, Kit was shifted to a slightly lower molecular weight, confirming altered glycosylation (Supplementary Figure S7a, top panels). The treatment did not however affect Kit’s localization and the activation of Akt, STAT5 and Erk (Supplementary Figures S7a and b). These results indicate that oncogenic Kit signaling on the Golgi is independent of glycosylation state.

### Activation of Src-family tyrosine kinases on the Golgi is essential for oncogenic Kit signaling

**Figure 6:**
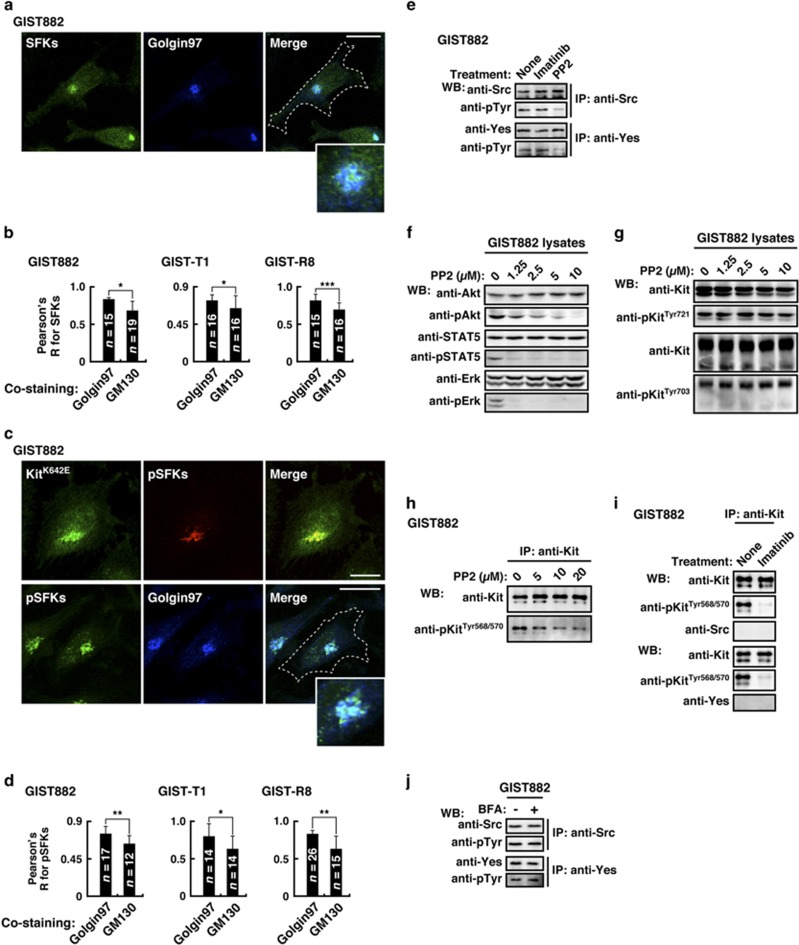
Kit(mut) requires SFKs on the Golgi for its downstream activation. (**a**–**d**) GIST882 cells were immuno-stained with the indicated antibody. Insets show magnified images of the perinuclear region. Dashed lines indicate cell borders. Bars, 20 μm. (**b**, **d**) The graph shows Pearson’s *R* between Golgi markers and SFKs or pSFKs. Results are means±s.d. from 12 to 26 cells. *P<0.05, **P<0.01, **P<0.001. Golgin97 (*trans*-Golgi marker), GM130 (*cis*-Golgi marker). (**e**) GIST882 cells were treated with 5 μM PP2 for 4 h. Anti-Kit immunoprecipitates were immunoblotted. (**f**, **g**) Immunoblots, lysates from GIST882 treated with PP2 for 4 h. (**h**–**j**) GIST882 cells were treated with (**h**) PP2 for 4 h, (**i**) 200 nM imatinib for 4 h, or (**j**) 1 μM BFA for 16 h. Immunoprecipitates were immunoblotted.

Previous studies showed that SFKs, such as Src, Yes, Lyn and Fyn localize to the Golgi through lipid modification, where they can initiate mitogenic signals.^49, 50^ Thus, we examined whether SFKs were involved in oncogenic Kit signaling. As shown in Supplementary Figure S8a, GIST-T1, GIST882 and GIST-R8 expressed Src and Yes. Although Src and Yes were undetectable by our immunofluorescence assay, anti-SFKs and anti-phosphorylated SFKs (anti-pSFKs) could visualize their intracellular location. SFKs predominantly localized to the *trans*-Golgi cisternae (Figures 6a and b and Supplementary Figure S8b). In addition, pSFKs localized to the *trans*-Golgi cisternae, where Kit(mut) accumulated (Figures 6c and d, and Supplementary Figure S8b), indicating that SFKs on the Golgi might participate in oncogenic Kit signaling.

Next, we investigated the effect of the inhibition of SFKs on Kit signaling. We confirmed that PP2 inhibited the autophosphorylation of Src and Yes (Figure 6e and Supplementary Figure S8c). SFK inhibition decreased the activation of Akt, STAT5 and Erk without affecting pKit[Tyr721] or pKit[Tyr703] (Figures 6f and g, and Supplementary Figure S8d). As shown in Figure 6h, the inhibition of SFKs decreased pKit[Tyr568/570]. In addition to PP2, the unrelated SFK inhibitor SU6656^50^ gave similar results (Supplementary Figure S8e and f). Our co-immunoprecipitation assays, however, showed that Src and Yes did not bind Kit(mut) (Figure 6i and Supplementary Figure S8g). These results suggest that SFKs have a role in the full functioning of Kit(mut) without any direct physical interaction. Also, BFA (a Golgi disruptor) and imatinib (a Kit inhibitor) did not affect the activation of SFKs (Figures 6e and j, and Supplementary Figures S8c and h), indicating that the activation of SFKs is independent of Kit(mut). In summary, Kit(mut) requires Golgi-localized SFK activity for oncogenic signaling (Supplementary Figure S8i). These results are consistent with our hypothesis that in GIST, the Golgi apparatus serves as a platform for oncogenic signaling.

Previous reports showed that SFKs regulate intra-Golgi transport.^50^ Thus, we tested whether SFKs have a role in retention of Kit(mut) on the Golgi. As shown in Supplementary Figure S9a, SFK inhibition did not affect the localization of Kit to the Golgi. Taken together with fact that SFK inhibition suppressed the PI3K–Akt pathway, STAT5 and Erk, it appears that these activations are not essential for Kit’s retention on the Golgi. In support of this, LY294002 (PI3K inhibition), Akt inhibitor and U0126 (Erk inhibition) had no effect on the localization of Kit to the Golgi (Supplementary Figures S9a and b). At present, the mechanism of Kit’s retention on the Golgi is not understood fully.

## Discussion

**Figure 7:**
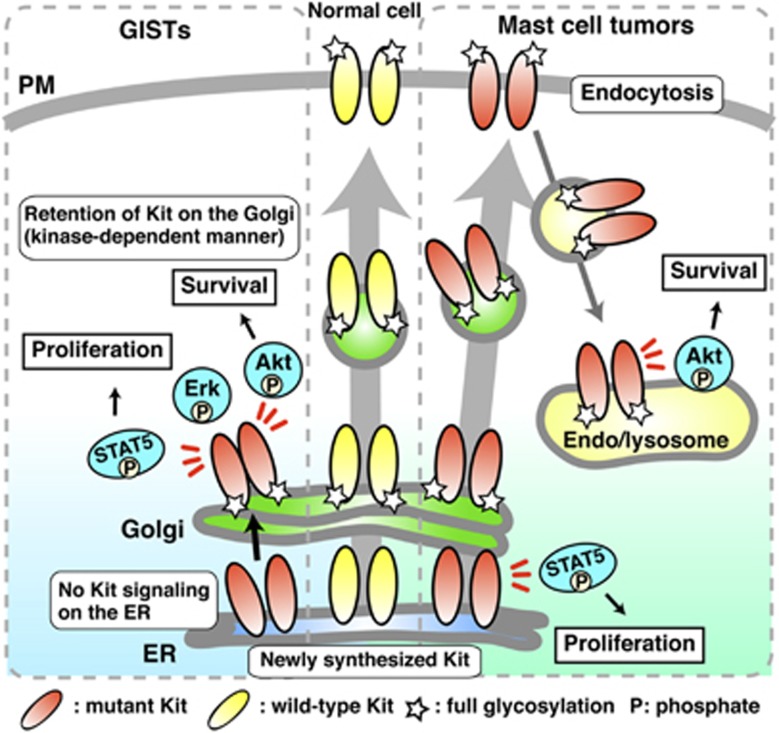
Model of oncogenic Kit signaling on intracellular compartments in GISTs and mast cell tumors. (Left, GISTs) Newly synthesized Kit(mut) traffics normally from the ER to the Golgi, then undergoes complex glycosylation as normal. After reacting the Golgi, Kit(mut) can activate the PI3K–Akt pathway, STAT5 and Erk. SFKs on the Golgi are needed for oncogenic Kit signaling. Activation of Kit in the wrong subcellular compartment, then prevents its export from Golgi to the PM. These mechanisms are common between imatinib-sensitive and imatinib-resistant Kit cases. (Right, mast cell tumors) Soon after synthesis, immature Kit is localized on the ER and activates STAT5. It then traffics to the PM along the secretory pathway. After Kit(mut) reaches the PM, it immediately moves to endolysosomes through endocytosis in a kinase activity-dependent manner. Kit–PI3K activates Akt specifically on endolysosomes.

In this study, we demonstrate that in GIST, Kit(mut) accumulates on the Golgi and that oncogenic Kit signaling occurs on the Golgi (Figure 7, left). Newly synthesized Kit(mut) traffics normally from the ER to the Golgi, then undergoes complex glycosylation as normal. After reacting the Golgi, Kit(mut) can activate the PI3K–Akt pathway, STAT5 and the Mek–Erk pathway. Activation of SFKs on the Golgi is needed for oncogenic Kit signaling. Activation of Kit in the wrong subcellular compartment then prevents its export from Golgi to the PM. These mechanisms are common between imatinib-sensitive and imatinib-resistant Kit(mut).

Aberrant accumulation of oncogenic receptors in the Golgi has been reported previously. FGFR3(K650E), a mutant found in multiple myelomas and skeletal dysplasia patients, is localized on the Golgi, then autophosphorylated,^51, 52^ and PDGFRα(mut) and Kit(mut) expressed in HEK293 or NIH3T3 localize to the Golgi region.^29, 35, 53^ H-Ras and SFKs, which are downstream molecules of RTKs, can initiate mitogenic signaling from the Golgi apparatus.^49, 50, 54, 55^ However, there has been no direct evidence that the mutant receptors cause oncogenic signaling on the Golgi apparatus. Here, we show that mutant receptors mis-localize in solid tumor using a panel of cancer tissues from GIST patients, and that Kit(mut) must localize to the Golgi to cause oncogenic activation of the PI3K–Akt pathway, STAT5 and Erk. Our study demonstrates in GISTs the pathological significance of the accumulation of mutant receptors in the Golgi for their signaling.

Recently, we reported that in mast cells, Kit(D814Y) activates the PI3K–Akt pathway and STAT5 on endo/lysosomes and the ER, respectively^34^ (Figure 7, right). In this study, Kit(D814Y) expressed in GIST cells, however, was autophosphorylated only on the Golgi apparatus. Namely, GIST and mastocytoma both show the same oncogenic Kit(mut) signaling mechanism, but the signaling platforms are different. These results are consistent with the idea that there may be different regulatory mechanisms of oncogenic signaling initiated from organelles where Kit is localized.

In considering putative mechanisms, in GIST, Kit(mut) accumulated on the Golgi in a manner dependent only on its kinase activity, and independent of downstream activation. Inhibitors of downstream activation, such as Ly294002 and U0126, do not block Kit’s phosphorylation and association with other proteins, suggesting that feature of the phosphorylated Kit(mut) complex, such as shape, size, and/or charge, is responsible for its retention in the Golgi apparatus. On the other hand, in mastocytoma, Kit(mut) can move from the Golgi normally.^34^ Further analyses of differences of trafficking machinery between mastocytoma and GIST may explain the Golgi retention of Kit(mut).

Mutations in Kit’s juxta-membrane domain or tyrosine kinase domain 1 (TKD1) are common in GISTs,^11, 23, 24^ but rare in human neoplastic mast cell disorders.^14^ Neoplastic mast cells commonly have a mutation elsewhere, in the activation loop of Kit’s tyrosine kinase domain 2 (TKD2).^14^ GIST-type Kit may be insufficient for transforming mast cells. A previous study on Ba/F3 cells showed that the TKD1 mutant but not the juxta-membrane domain mutant can suppresses the expression of SH2-containing inositol-5'-phosphatase 1 (SHIP1)^56^ that inhibits the PI3K–Akt pathway,^57^ suggesting that different Kit mutants use different signaling pathways. The TKD2 mutant may have novel functions, which GIST-type Kit does not have. TKD2 mutant may lead mast cells to autonomous proliferation, not only through SHIP1 suppression, but also through other signal pathways different to those seen in GIST. Forced expression of each mutant in mast cells and analyses of signaling will help us to understand the precise mechanism.

Phosphatidylinositol-3,4,5-triphosphate (PI(3,4,5)P_3_), which is necessary for Akt activation, is believed to be generated only at the PM by Kit–PI3K.^3, 4, 9^ This seems to be inconsistent with our observation that Kit activates Akt through PI3K on the Golgi. However, previous studies showed that PI(4,5)P_2_, a substrate of PI3K, exists on the Golgi,^58, 59, 60^ supporting our hypothesis that Golgi-localized Kit–PI3K can activate Akt. On the other hand, in the ER, PI(4,5)P_2_ is converted mainly to PI(4)P,^59^ so ER-localized Kit–PI3K cannot activate Akt. Further studies will be required to understand the mechanism by which the Kit–PI3K complex activates Akt selectively on the Golgi apparatus.

Tyrosine kinase inhibitors (TKIs) and antibodies have been widely used for blocking signaling from RTKs.^61, 62^ Our study implicates that mutant RTKs, such as Kit(mut) and PDGFRα(mut), can escape from antibodies because the mutants are trapped inside cells. Since treatment with TKIs causes mutant RTKs to remain at the PM through inhibiting mislocalization of the mutants,^29, 34, 35, 63, 64^ TKIs might also enhance the activity of antibody in blocking oncogenic signaling. From this point of view, combined therapy with TKIs and antibody seems attractive.

Imatinib is efficacious in most patients with primary GISTs harboring Kit mutations.^23, 24, 25^ However, two major problems arise in this targeting therapy. First, resistance to the drug appears with prolonged use. Most imatinib-resistant cases have a secondary Kit mutation in the kinase domain, and then lose sensitivity to the drug.^23, 26, 27^ In other cancers with RTK(mut), resistance to TKIs develops in a manner similar to imatinib-resistant Kit.^65^ In this study, we showed that blockade of Kit trafficking from the ER suppresses its oncogenic signaling. Importantly, its blockade could suppress oncogenic Kit signaling regardless of resistance to TKI drugs. These results suggest that blockade of trafficking may be a new strategy for inhibition of TKI-resistant RTKs. A second concern with imatinib treatment is its side effects. A previous study reported that patients with a duplication in the Kit extracellular domain require a higher dosage of imatinib.^13, 66^ However, side effects such as nausea and anemia limit dosage. Recent studies showed that imatinib accumulates mainly in lysosomes but not in the Golgi.^67, 68^ Golgi-targeting of imatinib by chemical modifications might improve therapeutic efficacy and reduce side effects. Since the cancer-causing mutants of EGF-R, Met, Flt3 and gp130 also initiate their signals from organelles,^64, 69, 70, 71, 72, 73^ intracellular delivery of TKIs to signaling platforms will open new fields for targeting therapy.

In conclusion, we show that oncogenic Kit signals from the Golgi are essential for the autonomous proliferation of GIST cells. These findings provide new insights not only into the pathogenic role of Kit(mut) but also into targeting therapy. Improper trafficking and aberrant signaling are frequent features of mutant RTKs. Our findings shed light on the significance of the spatial organization of this oncogenic signaling, and suggest strategies for the improvement of therapy.

## Materials and methods

### Cell culture

GIST-T1 (Cosmobio, Tokyo, Japan) and HeLa (American Type Culture Collection, Manassas, VA, USA) were cultured at 37 °C in DMEM supplemented with 10% FCS, penicillin, streptomycin and glutamine (Pen/Strep/Gln). An imatinib-resistant cell line GIST-R8 was cultured in DMEM supplemented with 10% FCS, Pen/Strep/Gln and 1 μM imatinib.^37^ GIST882 cells were cultured in RPMI1640 medium supplemented with 10% FCS and Pen/Strep/Gln. HMC-1.2 cells were cultured as previously described.^34^

### Chemicals

Imatinib (Cayman Chemical, Ann Arbor, MI, USA), PKC412, SU6656 (Santa Cruz Biotechnology, Dallas, TX, USA), Akt inhibitor VIII, LY294002, U0126, PP2 (Millipore, La Jolla, CA, USA), Pitstop2 (Abcam, Cambridge, UK) and filipin (Sigma, St Louis, MO, USA) were dissolved in dimethyl sulfoxide. Bafilomycin A1, brefeldin A (Sigma) and monensin (Biomol, Exeter, UK) were dissolved in ethanol. Swainsonine (Wako, Osaka, Japan) was dissolved in methanol.

### Antibodies

The following antibodies were purchased: Kit (M-14), STAT5 (C-17), Erk2 (K-23), Src (Src2) and from Santa Cruz Biotechnology; Kit[pTyr719], Kit[pTyr703], Akt (40D4), Akt[pT308] (C31E5E), STAT5[pTyr694] (D47E7), PDGFRα (D13C6), Erk[pThr202/pTyr204] (E10), golgin97 (D8P2K) and Src[pY416] (D49G4) from Cell Signaling Technology (Danvers, MA, USA); p85, Kit[pTyr568/570], pTyr (4G10) and Src (327) from Millipore; golgin97 (CDF4) and calnexin (AF18) from Thermo Scientific Pierce (Rockford, IL, USA); calnexin from Enzo (Farmingdale, NY, USA); GM130 (35), Yes (1) and Fyn (25) from BD Transduction Laboratories (Franklin Lakes, NJ, USA); LAMP1 from Sigma and Kit (104D2) from Biolegend (San Diego, CA, USA). Horseradish peroxidase-labeled secondary antibodies were purchased from the Jackson Laboratory (Bar Harbor, MA, USA). Alexa Fluor-conjugated secondary antibodies were obtained from Molecular Probes (Eugene, OR, USA).

### Cancer tissue samples from GIST patients

Cancer tissue samples were collected from patients with GIST in the Department of Surgery, Osaka University Hospital and the Department of Diagnostic Pathology, Osaka Police Hospital. The protocol for the collection and use of the tissue samples was approved by the ethics committees of Osaka University Hospital (14154-2) and Tokyo University of Science (15005). Written informed consent was obtained from each patient before surgery. Patient identifiers were unavailable to investigators.

### Immunofluorescence confocal microscopy in GIST cell lines and cancer tissue samples

Cells cultured on poly-L-lysine-coated coverslips were fixed with 4% paraformaldehyde for 20 min at room temperature. Fixed cells were permeabilized and blocked for 30 min in PBS supplemented with 0.1% saponin and 3% BSA, and then incubated with a primary and secondary antibody for 1 h each. To stain for SFKs, cells were fixed with methanol for 10 min at −20 °C, and 5% skimmed milk was used for blocking. For staining the extracellular domain of Kit, cells were incubated with anti-Kit(104D2) in the presence of 0.1% NaN_3_ at 4 °C for 1 h without permeabilization. Subsequently, cells were incubated with secondary antibody and 10 μM Höechst33342 for 1 h, and then fixed with 4% paraformaldehyde. To visualize lysosomes, cells were incubated for 1 h with 100 nM LysoTracker Red (Molecular Probes, Eugene, OR, USA). GIST-R8 cells were cultured in the presence of 1 μM imatinib before fixation. For immunofluorescence analysis in cancer tissue samples, paraffin-embedded blocks were cut into 4 μm sections using a microtome. The sections were deparaffinized in xylene, rehydrated with decreasing proportions of alcohol to water, and then boiled in 1 mM EDTA (pH 8.0) for 15 min by a microwave oven for antigen retrieval. Immunostaining was performed as above. Confocal images were obtained with a Fluoview FV10i laser scanning microscope with an × 60 1.20 NA water-immersion objective (Olympus, Tokyo, Japan). Composite figures were prepared with Photoshop Elements 10 and Illustrator CS6 software (Adobe, San Jose, CA, USA). Pearson’s R were calculated with NIH ImageJ 1.48v software.

### Immunoprecipitation and western blotting

Lysates from 0.1 to 1.5 × 10^6^ cells were prepared in SDS–PAGE sample buffer or NP-40 lysis buffer (50 mM HEPES, pH 7.4, 10% glycerol, 1% NP-40, 4 mM EDTA, 100 mM NaF, 1 μg/ml aprotinin, 1 μg/ml leupeptin, 1 μg/ml pepstatin A, 1 mM PMSF and 1 mM Na_3_VO_4_). Immunoprecipitation was performed at 4 °C for 5 h using protein G pre-coated with antibody. Immunoprecipitates were dissolved in SDS-PAGE sample buffer, subjected to SDS–PAGE, and electro-transferred onto PVDF membranes. Immunodetection was performed by ECL (PerkinElmer, Waltham, MA, USA). Results were analyzed with an LAS-3000 image analyzer with Science Lab software (Fujifilm, Tokyo, Japan).

### Plasmid DNA and transfection

Mouse cDNAs encoding Kit were carboxy-terminally tagged with GFP as described.^34^ Transient transfection was performed using Fugene HD transfection reagent (Roche, Rotkreuz, Switzerland).

### Analysis of protein glycosylation

Following the manufacturer’s instructions (New England Biolabs, Ipswich, MA, USA), NP-40 cell lysates were treated with endoglycosidases for 1 h at 37 °C. The reactions were stopped with SDS–PAGE sample buffer, products were resolved by SDS–PAGE and immunoblotted.

### Statistical analyses

For statistical analysis, experiments were repeated as three biological replicates. Differences between two or more groups were analyzed by the two-tailed Student’s *t*-test or one-way analysis of variance followed by the Dunnett’s multiple comparison *post-hoc* test, respectively. All statements of significant differences showed a 5% level of probability.

## Figures and Tables

**Table 1:** Summary of the localization of Kit and PDGFRα in cancer tissue from GIST patients

*Mutation*		n	*Localization*	
			*Kit*	*PDGFRα*
Kit	Ex9	4	Golgi	–
Kit	Ex11	7	Golgi	–
Kit	Ex17	2	Golgi	–
–	–	7	PM	–
PDGFRα	Ex18	3	ND	Golgi
Kit	Ex11+Ex13	1	Golgi	–
Kit	Ex11+Ex17	3	Golgi	–
Kit	Ex9+Ex17	1	ND	–

Abbreviations: Ex, exon; ND, Kit expression was not detected by our immunofluorescence microscopy; PM, plasma membrane.

## Abbreviations

BFA: brefeldin A
ER: endoplasmic reticulum
GIST: gastrointestinal stromal tumor
Kit(mut): mutant Kit
PDGFR: platelet-derived growth factor receptor
PI3K: phosphatidylinositol 3-kinase
PI(3,4,5)P_3_: phosphatidylinositol-3,4,5-triphosphate
PM: plasma membrane
RTK: receptor tyrosine kinase
SFKs: Src-family tyrosine kinases
STAT: signal transducer and activator of transcription
TKI: tyrosine kinase inhibitor
wt: wild-type.
