# Supplementary material for: Oncogenic signaling by Kit tyrosine kinase occurs selectively on the Golgi apparatus in gastrointestinal stromal tumors
Source: Oncogene. 2017 Feb 13;36(26):3661–72. doi: 10.1038/onc.2016.519 (PMC5500841; doi:10.1038/onc.2016.519)
Supplement: Supplementary Information [file onc2016519x1.pdf]

Supplementary Data

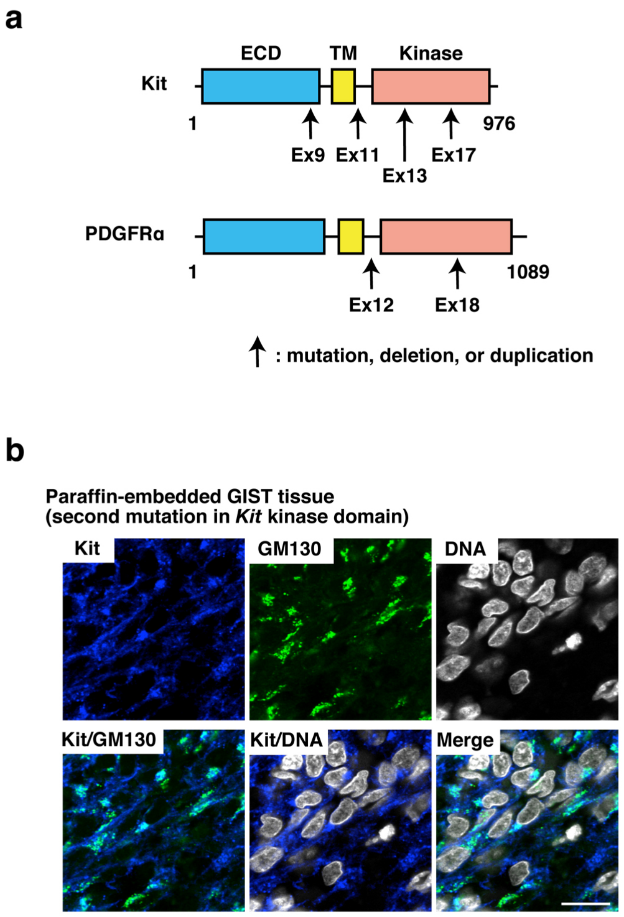

Supplementary Figure S1. Mutations in *Kit* and *PDGFRα* in GISTs. (**a**) Schematic representations of Kit and PDGFRα showing the extracellular domain (ECD), the transmembrane domain (TM), and the kinase domain. Arrows indicate the positions of mutation, deletion, or duplication, which lead the receptors to become constitutively active. Ex, exon. (**b**) Paraffin-embedded cancer tissue from an imatinib-resistant GIST patient bearing a secondary Kit mutation was stained for Kit (blue), GM130 (Golgi marker, green), and DNA (white). Bar, 20 μm.

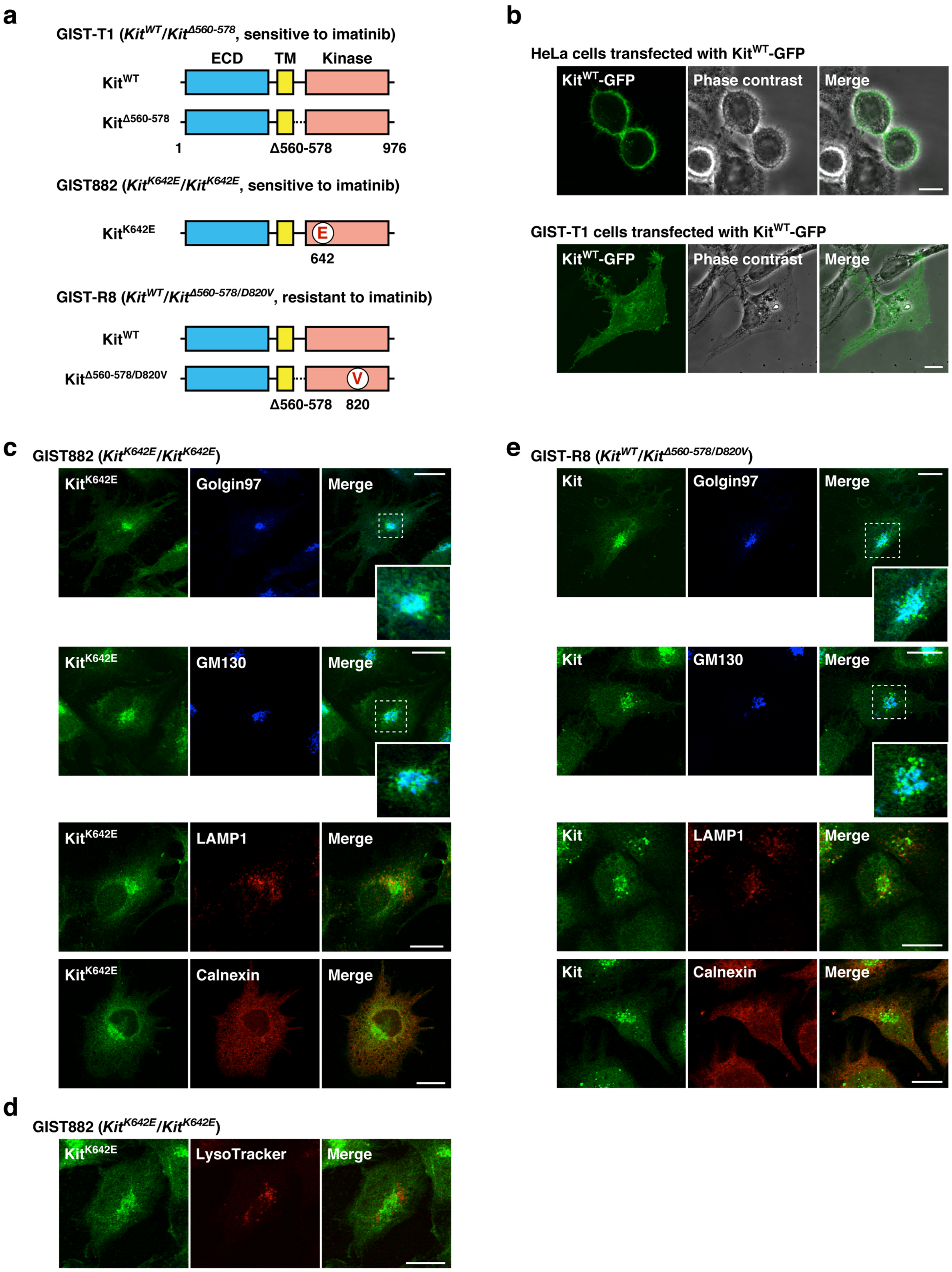

Supplementary Figure S2. In GIST cell lines, Kit(mut) localizes preferentially on the Golgi apparatus. (**a**) Schematic representations of wild-type Kit (Kit^{WT}), and constitutively active Kit mutants ($Kit^{\Delta560\text{-}578}$, Kit^{K642E}, $Kit^{\Delta560\text{-}578/D820V}$) are shown. Dashed lines indicate an in-frame deletion in the juxtamembrane domain. ECD, extracellular domain; TM, transmembrane domain. (**b**) GIST-T1 and HeLa were transfected with Kit^{WT}-GFP for 24 h. Phase contrast images are shown. Bars, 10 μm. GFP, green fluorescent protein. (**c**-**e**) GIST882 (**c** and **d**) and GIST-R8 (**e**) were immuno-stained with the indicated antibody. Insets show magnified images of the boxed areas. Bars, 20 μm. Golgin97 (*trans*-Golgi marker), blue; GM130, (*cis*-Golgi marker), blue; calnexin (ER marker), red; LAMP1 (endo/lysosome marker), red. Lysosomes were visualized with LysoTracker Red (**d**).

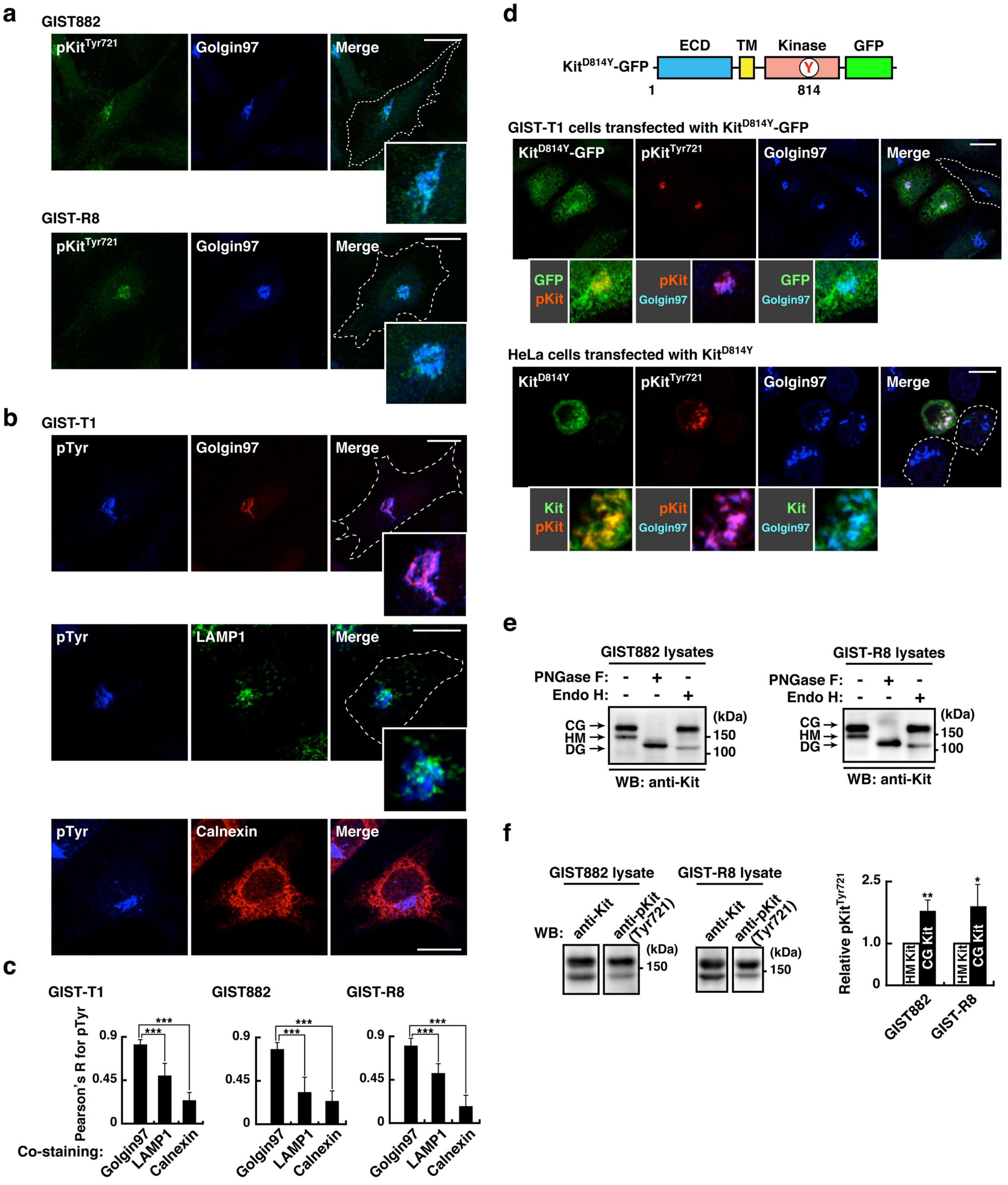

Supplementary Figure S3. In GIST cell lines, Kit's autophosphorylation occurs mainly on the Golgi apparatus. (**a** and **b**) GIST882, GIST-R8, or GIST-T1 were immuno-stained with anti-pKit^{Tyr721} (**a**), anti-pTyr (**b**) and the indicated antibody. Insets show magnified images of the perinuclear region. Dashed lines indicate cell borders. Bars, 20 μm. (**c**) Pearson's R correlation coefficients (Pearson's R) were calculated from intensity analysis of pTyr *vs*. organelle markers. Results are means ± s.d. (n = 14~35). ***P < 0.001. Golgin97 (*trans*-Golgi marker), blue or red; LAMP1 (endo/lysosome marker), green; calnexin ER marker, red. pKit^{Tyr721}, phosphorylation at Tyr721 in Kit; pTyr, phosphotyrosine. (**d**) GIST-T1 and HeLa were transfected with Kit^{D814Y}-GFP and stained with the indicated antibody. Insets show magnified images of the perinuclear region. Dashed lines indicate untransfected cells. Bars, 20 μm. GFP, green fluorescent protein. (**e**) Lysates from GIST882 and GIST-R8 were treated with peptide N-glycosidase F (PNGase F) or endoglycosidase H (endo H), then immunoblotted. CG, complex-glycosylated form; HM, high mannose form; DG, deglycosylated form. (**f**) Levels of phosphorylation of the complex-glycosylated form of Kit are expressed relative to those of the high mannose form of Kit. Results are means ± s.d. (n = 3). *P < 0.05, **P < 0.01.

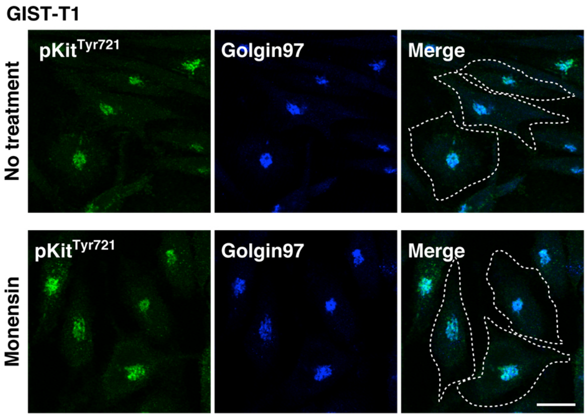

Supplementary Figure S4. Kit(mut) accumulates on the Golgi during the early secretory pathway but not after endocytosis. GIST-T1 cells treated with 250 nM monensin (blocks Golgi export) for 24 h were stained with anti-pKit^{Tyr721} (green) and anti-golgin97 (*trans*-Golgi marker; blue). Dashed lines indicate cell borders. Bars, 20 μm.

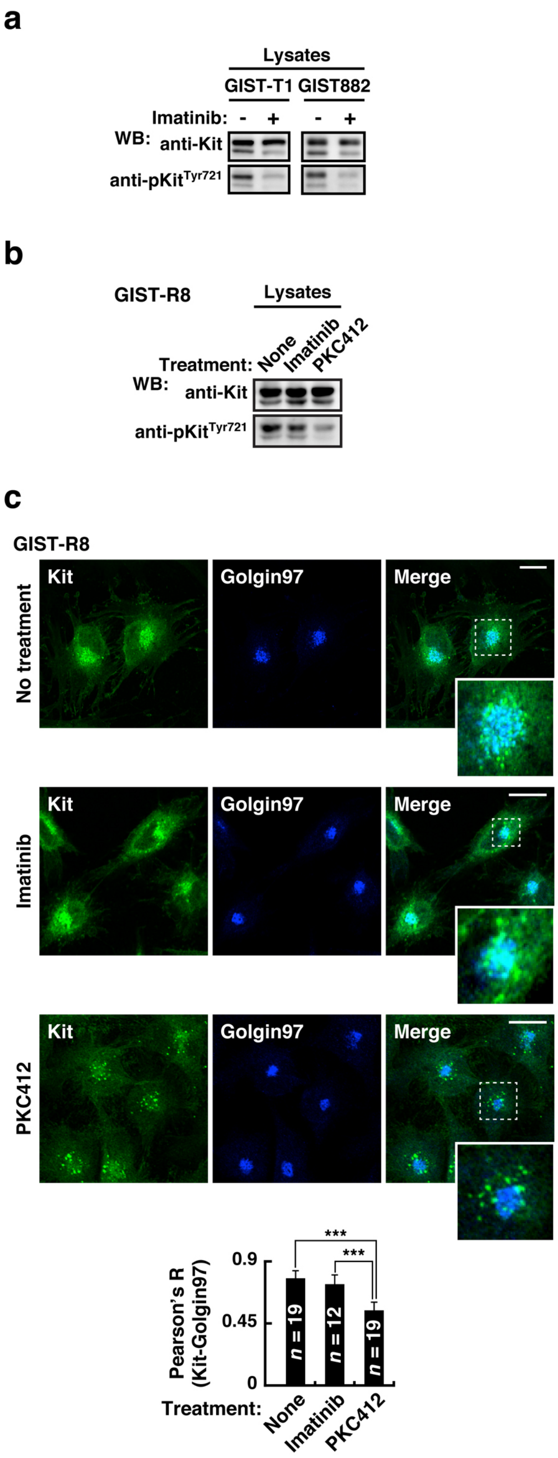

Supplementary Figure S5. Kit's activity prevents Kit's export from the Golgi apparatus. (**a**) GIST-T1 and GIST882 were treated with 200 nM imatinib (Kit inhibitor) for 4 h. Lysates were immunoblotted with the indicated antibody. (**b**) Immunoblots, lysates from GIST-R8 cells treated with 1 μM imatinib or 1 μM PKC412 (inhibits imatinib-resistant Kit) for 4 h. (**c**) GIST-R8 cells were treated with 1 μM imatinib or 1 μM PKC412 for 4 h then stained with anti-Kit (green) and anti-golgin97 (Golgi marker, blue). Insets show magnified images of the boxed area. Bars, 20 μm. The graph shows Pearson's R correlation coefficients between Kit and golgin97. Results are means ± s.d. (n = 12~19). ***$P < 0.001$.

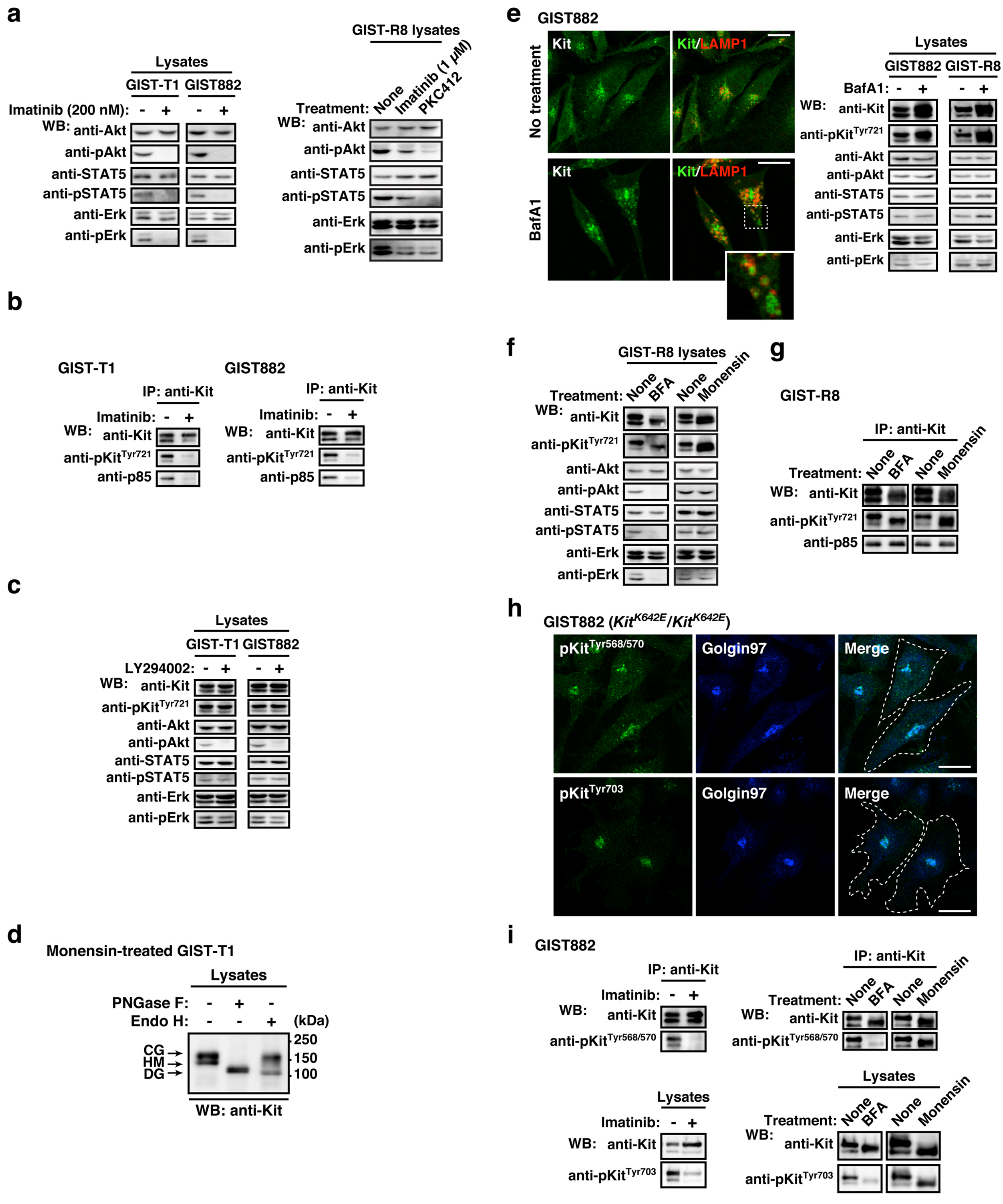

Supplementary Figure S6. Kit(mut) on the Golgi signals and activates the PI3K-Akt pathway, STAT5, and Erk. (**a**) GIST-T1, GIST882, or GIST-R8 were treated with 200 nM or 1 μM imatinib, or 1 μM PKC412 for 4 h. Lysates were immunoblotted with the indicated antibody. Phosphorylated proteins are presented as pKit, pAkt, pSTAT5, and pErk. (**b**) GIST-T1 and GIST882 were treated with 200 nM imatinib (Kit inhibitor). Anti-Kit immunoprecipitates were immunoblotted. (**c**) GIST-T1 and GIST882 were treated with 5 μM LY294002 (PI3K inhibitor) for 4 h. Lysates were immunoblotted. (**d**) GIST-T1 cells were treated for 24 h with 250 nM monensin (blocks Golgi export). Glycosylation was assayed as for Figure 3d. CG, complex-glycosylated form; HM, high mannose form; DG, deglycosylated form. (**e** and **f**) GIST882 or GIST-R8 were treated with (**e**) 100 nM bafA1 (bafilomycin A1; blocks endo/lysosomal trafficking) for 24 h, (**f**) 1 μM BFA (brefeldin A; blocks ER export to the Golgi) for 16 h, or 250 nM monensin (blocks Golgi export) for 24 h. Immunofluorescence images and immunoblots are shown. (**g**) GIST-R8 cells were treated with BFA or monensin as above. Anti-Kit immunoprecipitates were immunoblotted. (**h**) GIST882 cells were immuno-stained for pKit$^{Tyr568/570}$, pKit^{Tyr703} (green), and golgin97 (Golgi marker, blue). Dashed lines indicate cell borders. Bars, 20 μm. (**i**) Anti-Kit immunoprecipitates from GIST882 treated with (left) 200 nM imatinib for 4 h, (right) 1 μM BFA for 16 h, or 250 nM monensin for 24 h were immunoblotted.

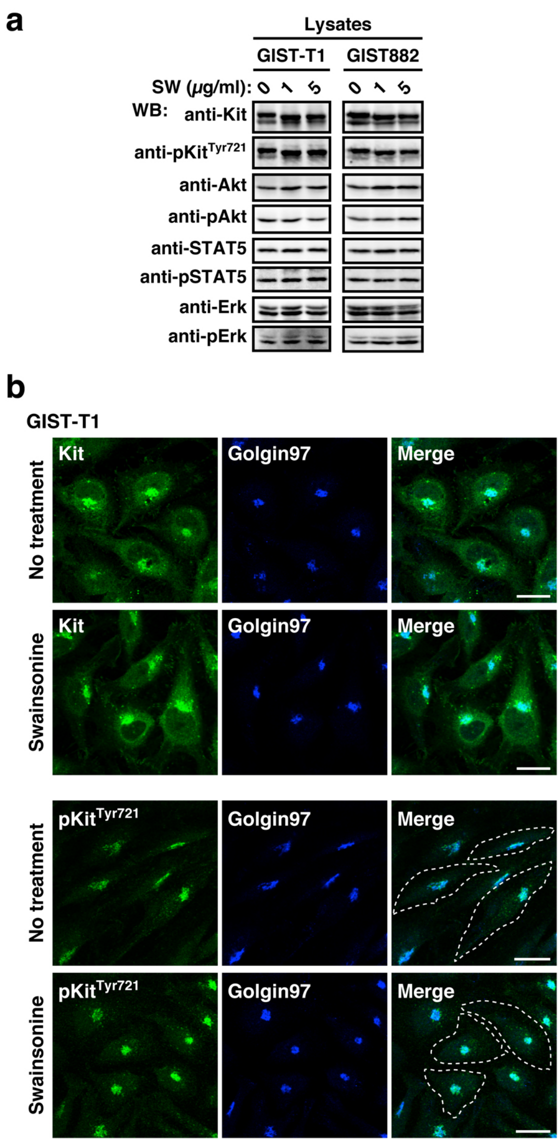

Supplementary Figure S7. Change of Kit's glycosylation states by swainsonine does not affect Kit's localization and oncogenic signaling. (**a**) Immunoblots, lysates from GIST cells treated with swainsonine (changes glycosylation states of proteins) for 24 h. (**b**) GIST-T1 cells were treated with 5 μg/ml swainsonine for 24 h, then immuno-stained. Dashed lines indicate cell borders. Bars, 20 μm.

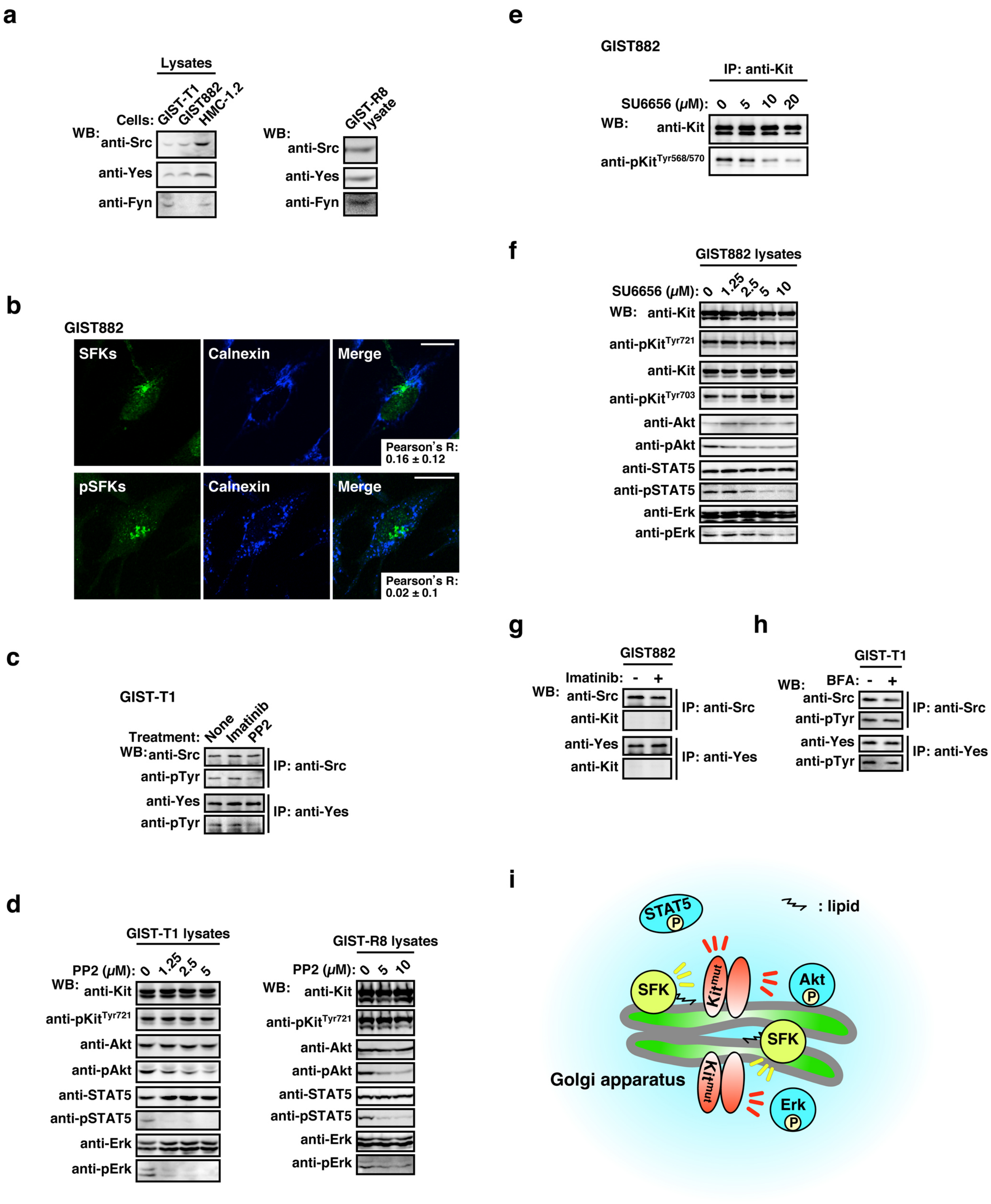

Supplementary Figure S8. Kit(mut) requires SFKs on the Golgi for its downstream activation. (**a**) Immunoblots, lysates from GIST-T1, GIST882, GIST-R8, and HMC-1.2 (human mast cell line). (**b**) GIST882 cells were immuno-stained with SFKs (green), anti-pSFKs (green), and anti-calnexin (ER marker, blue). Bars, 20 μm. Pearson's R correlation coefficients are shown. Results are means ± s.d. from 11~14 cells. SFKs, Src-family kinases; pSFKs, phosphorylated SFKs. (**c**) GIST-T1 and GIST882 were treated with 5 μM PP2 (SFK inhibitor) for 4 h. Anti-Src or anti-Yes immunoprecipitates were immunoblotted. (**d**) Immunoblots, lysates from GIST cells treated with PP2 for 4 h. (**e** and **f**) GIST882 cells were treated with SU6656 (SFK inhibitor) for 4 h. Lysates (**e**) and immunoprecipitates (**f**) were immunoblotted. (**g** and **h**) GIST882 or GIST-T1 were treated with (**g**) 200 nM imatinib (Kit inhibitor) for 4 h, or (**h**) 1 μM BFA (Golgi disruptor) for 16 h. Immunoprecipitates were immunoblotted. (**i**) Model of oncogenic signaling on the Golgi in GISTs. Note that Kit(mut) requires SFK activation on the Golgi for its full activation. P, phosphate.

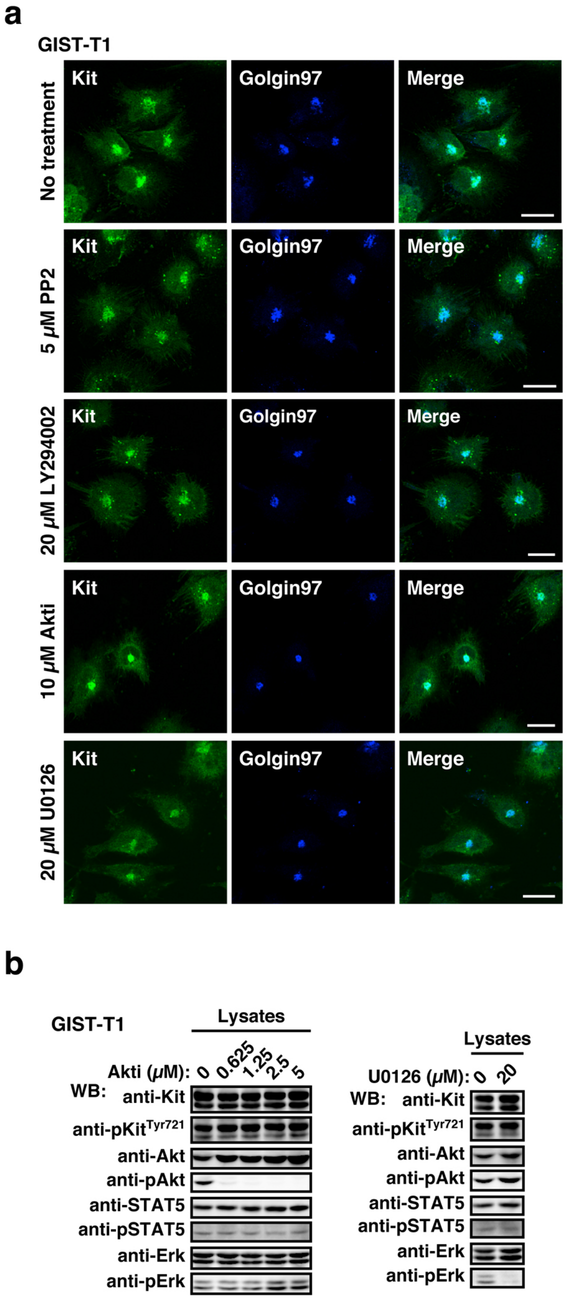

Supplementary Figure S9. Inhibition of SFKs, the PI3K-Akt pathway, and Erk does not affect the accumulation of Kit on the Golgi apparatus. (a) GIST-T1 cells were treated with 5 μM PP2 (SFK inhibitor), 20 μM LY294002 (PI3K inhibitor), 5 μM Akti (Akt inhibitor), or 20 μM U0126 (inhibits Erk). Cells were stained with the anti-Kit (green), anti-golgin97 (*trans*-Golgi marker; blue). Bars, 20 μm. (**b**) Immunoblots, lysates from GIST-T1 treated with 5 μM Akti or 20 μM U0126 for 4 h.
